# Quantitative activation-induced manganese-enhanced MRI reveals severity of Parkinson’s disease in mice

**DOI:** 10.1038/srep12800

**Published:** 2015-08-10

**Authors:** Satomi Kikuta, Yukiyo Nakamura, Yukio Yamamura, Atsushi Tamura, Noriyasu Homma, Yuchio Yanagawa, Hajime Tamura, Jiro Kasahara, Makoto Osanai

**Affiliations:** 1 Tohoku University Graduate School of Medicine, 2-1 Seiryo-machi, Aoba-ku, Sendai 980-8575, Japan; 2 CREST, Japan Science and Technology Agency, 4-1-8 Honcho, Kawaguchi 332-0012, Japan; 3 Research Fellow of the Japan Society for the Promotion of Science; 4 Graduate School and Faculty of Pharmaceutical Sciences, Institute of Biomedical Sciences, Tokushima University, 1-78 Shoumachi, Tokushima 770-8505, Japan; 5 Gunma University Graduate School of Medicine, 3-39-22 Showa-machi, Maebashi 371-8511, Japan

## Abstract

We demonstrate that activation-induced manganese-enhanced magnetic resonance imaging with quantitative determination of the longitudinal relaxation time (qAIM-MRI) reveals the severity of Parkinson’s disease (PD) in mice. We first show that manganese ion-accumulation depends on neuronal activity. A highly active region was then observed by qAIM-MRI in the caudate-putamen in PD-model mice that was significantly correlated to the severity of PD, suggesting its involvement in the expression of PD symptoms.

Manganese-enhanced magnetic resonance imaging (MEMRI) is being increasingly used for investigating neuronal pathways, brain architecture, and neuronal activities in the brain^1^,^2^. But quantitative comparisons of neuronal activity mapping of diseased vs. healthy animals have not been demonstrated. Manganese ions (Mn^2+^) can pass through opened voltage-dependent calcium channels^3^,^4^. Thus, in the presence of Mn^2+^ in the extracellular solution, highly active neurons should accumulate larger amounts of Mn^2+^ than weakly active neurons. Mn^2+^ shortens the longitudinal relaxation time (T_1_) of H^+^, making it an excellent MRI-detectable contrast agent^1^,^2^. Hence, MEMRI can non-invasively measure relative levels of neuronal activity and has been termed activation-induced MEMRI (AIM-MRI)^5^,^6^. Differential accumulation of Mn^2+^ in active and silent brain regions is generally assessed using T_1_-weighted images and quantified by the signal intensity. However, signal intensity is a relative value and can be unreliable in interanimal comparisons. Because Mn^2+^ concentration can be absolutely quantified by measuring the absolute T_1_ (or R_1_ = T_1_^−1^) value^7^, absolute determination of T_1_ in the entire brain volume may provide a powerful method for determining the topography of neuronal activity. However, it is not yet clear whether there is a direct relationship between neuronal activity and Mn^2+^ accumulation in the cell^1^.

Although the depletion of dopamine (DA) in the striatum is thought to cause changes in neuronal activities in the basal ganglia relevant to the symptoms of PD, the pathophysiological role of the striatum in PD is not fully elucidated^8^,^9^,^10^,^11^. Moreover, there is currently no definitive diagnostic test for PD, except at autopsy^12^. Therefore, to explore the application of AIM-MRI with quantitative T_1_ measurement (qAIM-MRI) as a useful non-invasive research and diagnostic tool, we used it to localize differences in neuronal activity in the brains of PD model mice and healthy controls.

## Results

**Figure 1:**
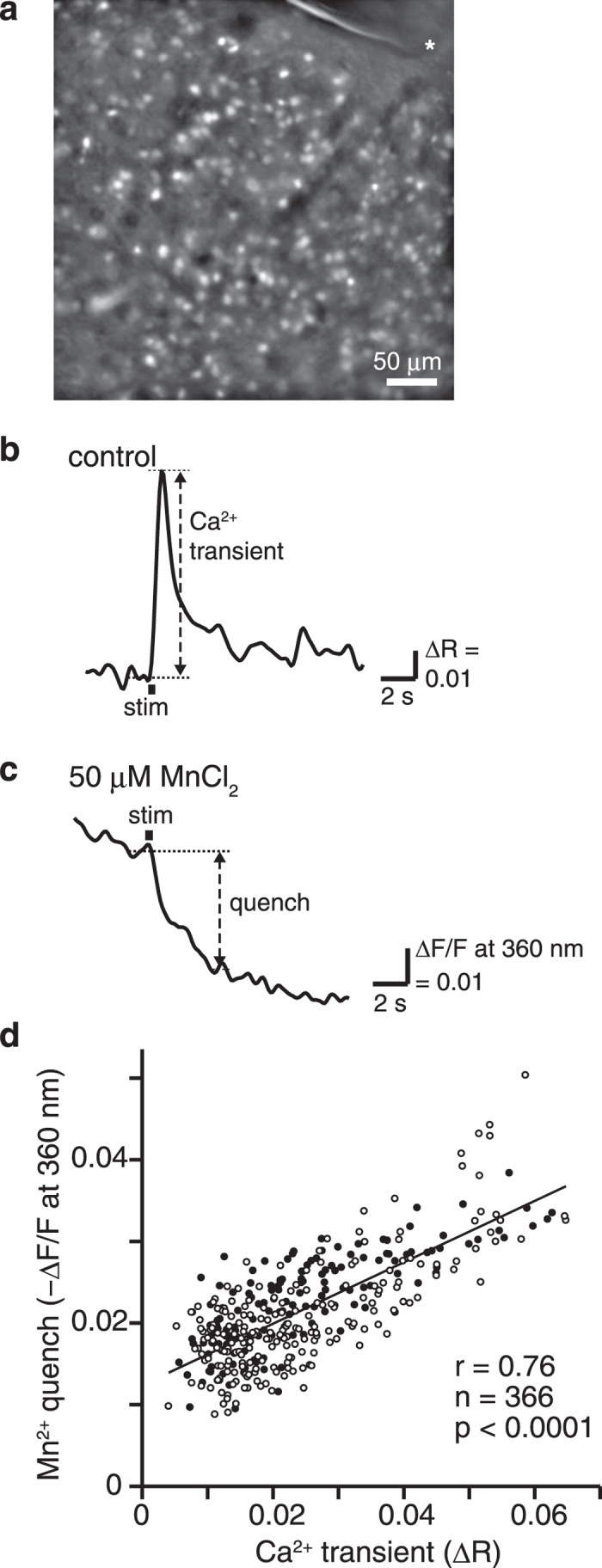
Intracellular Mn^2+^ accumulation was correlated with neuronal activity in striatal GABAergic neurons. (**a**) Fluorescence image of a striatal slice from a GAD67-GFP mouse. Striatal GABAergic neurons were identified by GFP fluorescence and ROIs were placed on the GFP-positive somata for quantification of the fluorescence changes. The tip of the stimulation electrode is indicated by asterisk (*). Scale bar, 50 μm. (**b**,**c**) Typical time course of the [Ca^2+^]_i_ transient evoked by 20 pulses of 50-Hz stimulation before (**b**) and following 50 μM MnCl_2_ administration (**c**) obtained from the same cell. Dashed arrows indicate amplitude of the [Ca^2+^]_i_ transient (**b**) or the amount of the Mn^2+^ quench (**c**). Stimulation period is indicated by thick horizontal line. (**d**) Comparison of the amplitude of the [Ca^2+^]_i_ transient and the amount of Mn^2+^ quench of the fluorescence at 360 nm, when 20 pulses at 20 Hz (solid circle) or 50 Hz stimuli (open circle) were applied (*n* = 366 cells). *r*: Pearson’s correlation coefficient.

We first confirmed that intracellular Mn^2+^ accumulation is correlated with neuronal activity in individual cells using calcium (Ca^2+^) imaging in mouse brain slices. By stimulating the corticostriatal fiber tract in slices and measuring intracellular Ca^2+^ concentration ([Ca^2+^]_i_) in individual striatal GABAergic neurons, we confirmed that the amplitude of the [Ca^2+^]_i_ transient elevation (change in Fura-2 LR fluorescence emission) changed with differing stimulation frequency^13^,^14^ and could, thus, be treated as the index of neuronal activity (Supplementary Fig. S1). As Mn^2+^ quenches Fura-2 LR fluorescence emission, the amount of fluorescence quench reflects intracellular Mn^2+^ accumulation. Mn^2+^ accumulation in striatal GABAergic neurons (Fig. 1a) was determined by comparing [Ca^2+^]_i_ transients with fluorescence quench induced by 20 pulses of 20-Hz or 50-Hz stimulation to the corticostriatal fiber tract before and after administration of 50 μM MnCl_2_ in slice perfusates (Fig. 1b,c). A strong positive linear correlation was observed between amplitudes of [Ca^2+^]_i_ transients and amounts of Mn^2+^ quench of the fluorescence in GABAergic neurons (*r* = 0.76, *P* < 0.0001, *n* = 366; Fig. 1d), indicating that intracellular Mn^2+^ accumulation is correlated with neuronal activity. The Mn^2+^ accumulation was also correlated with stimulus evoked Ca^2+^ elevation in astrocytes (supplementary Fig. S2).

In another preliminary experiment, we dissolved 2% (wt/vol) agarose gels in artificial intracellular solution containing different amounts of MnCl_2_, and showed that the longitudinal relaxation rate R_1_ acquired via rapid acquisition with relaxation enhancement (RARE) with variable repetition time (TR) pulse sequence (RARE-VTR), was proportional to the concentration of MnCl_2_ (R_1_ = 5.35 [Mn^2+^] (mM) + 0.32, *P* < 0.0001; Supplementary Fig. S3). The relaxivity of Mn^2+^ was r_1_ = 5.35 ± 0.32 mM^−1^ s^−1^.

We then applied qAIM-MRI to map the location of neuronal activity changes in PD model mice produced by 1-methyl-4-phenyl-1,2,3,6-tetrahydropyridine hydrochloride (MPTP) intoxication (20 mg kg^−1^, i.p., four times at 2-h intervals)^15^, compared with healthy control mice. These T_1_ maps were quantitatively assessed by statistical parametric mapping (SPM).

**Figure 2:**
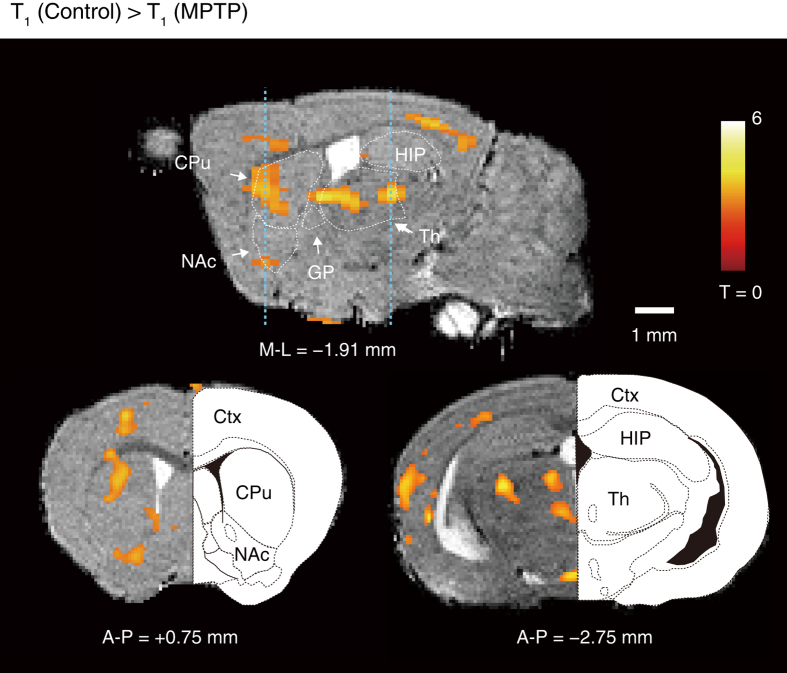
The active regions in PD compared with control mice quantified by qAIM-MRI. Regions with significant shortening of T_1_ in PD mice are indicated by pseudo-colored regions over the T_2_-enhanced brain image template in sagittal (upper) and coronal (lower) planes (*n*_control_ = 6 mice, *n*_PD_ = 6 mice). Regions were defined by brain atlas alignment to MRI image: those regions defined in text, plus nucleus accumbens (NAc), globus pallidus (GP), and hippocampus (HIP). The large colored regions were observed in CPu, Ctx, and Th. T: t-value.

One or two weeks after MPTP or saline injection, MnCl_2_ was injected (0.2 mmol kg^−1^, i.p.) twice at 24-h intervals (Supplementary Fig. S4). To visualize regions with significantly elevated activity in PD vs. control mice, the voxels with significant T_1_-shortening in the MPTP (*n* = 6) vs. control (*n* = 6) mice were defined as those with *P*-values below 0.025 by SPM analysis (Fig. 2). Within the basal ganglia, the caudate-putamen (CPu) showed significant shortening of T_1_, with a t-value of Student’s *t*-test of 3.21 (*P* < 0.01) at coordinates medial-lateral (M-L) = −1.91, anterior-posterior (A-P) = 0.75, and dorsal-ventral (D-V) = 2.93 (mm), indicating that the activity in the CPu increased in PD mice compared with control mice. In the cortex (Ctx) and the thalamus (Th), the regions exhibiting significant T_1_ shortening were observed (Fig. 2). The t-values of Student’s *t*-test were 3.51 (*P* < 0.01) at coordinates M-L = −1.77, A-P = 0.75 and D-V = 1.1 (mm), and 3.08 (*P* < 0.02) at coordinates M-L = −0.4, A-P = −2.75 and D-V = 3.76 (mm) in the sensorimotor cortex and the parafascicular nucleus of the thalamus, respectively.

**Figure 3:**
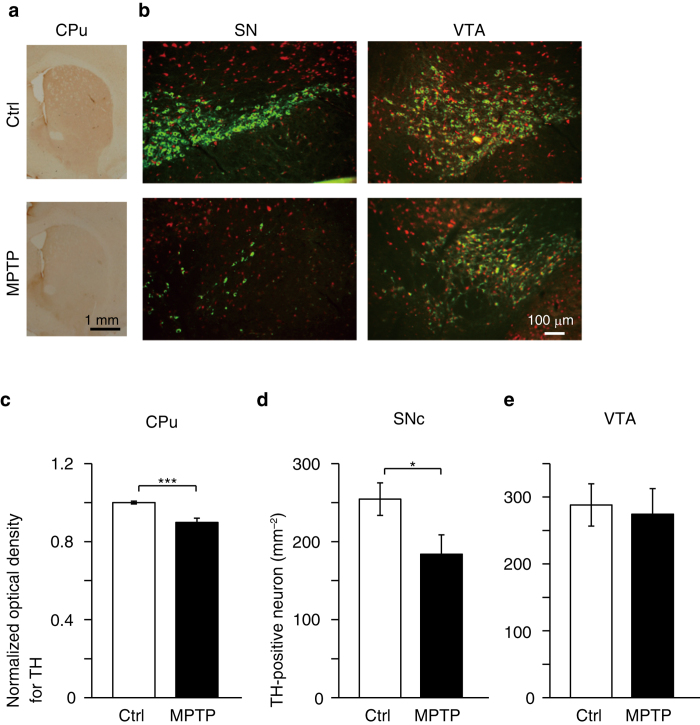
MPTP-induced loss of TH-positive fibers in CPu and of TH-positive neurons in SNc. (**a**) Photomicrographs of TH-immunostained sections from the striatum (CPu) of control (Ctrl, upper) and MPTP-treated (MPTP, lower) mice. Scale bar, 1 mm. (**b**) Immunofluorescent staining of TH (green) and NeuN (red) in slices from SNc (left) and VTA (right) of control and MPTP mice. Scale bar, 100 μm. (**c**) Normalized optical densities for TH-immunoreactivity in CPu of control and MPTP mice. The optical density of TH-immunoreactivity was normalized by the average optical density from control mice in each batch. ***P < 0.002. (**d**,**e**) The number of neurons expressing TH in SNc (**d**) and VTA (**e**) of control and MPTP mice. *P < 0.05. (**c**–**e**) *n*_ctrl_ = 6 mice, *n*_MPTP_ = 6 mice.

Finally, we analyzed the correlation between striatal neuronal activity obtained by qAIM-MRI and tyrosine hydroxylase (TH)-immunoreactivity in the striatum, which is correlated to motor performance in PD model mice^16^. Eleven to twenty days after MPTP injection, TH-positive fibers in the CPu of the MPTP mice were reduced to 89.8 ± 2.2% of those in controls (*P* < 0.002; Fig. 3a,c). The number of TH-positive cells in the substantia nigra pars compacta (SNc) of the MPTP mice was reduced to ~72% (control: 254 ± 21 vs. MPTP: 184 ± 25 mm^–2^; Fig. 3b,d). No significant TH-positive cell loss was observed in the ventral tegmental area (VTA) (control: 288 ± 32 vs. MPTP: 274 ± 38 mm^–2^; Fig. 3b,e). Thus, our MPTP administration resulted in a degeneration of DA neurons in the SNc resembling that of Parkinson’s disease, but the severity was mild^17^, as the loss of TH-positive neurons, though significant, was relatively small.

**Figure 4:**
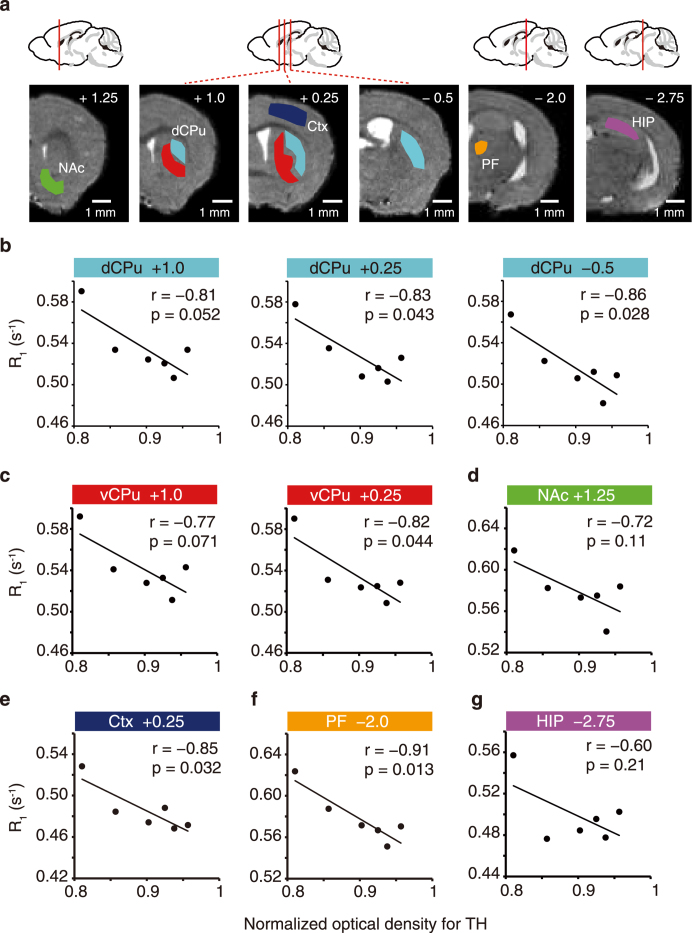
R_1_ in CPu represents the severity of PD. (**a**) ROIs were defined by brain atlas alignments to MRI images. The identifying number in each image indicates the A-P distance from bregma; slice positions are illustrated above the images. Scale bar, 1 mm. (**b–g**) Comparisons of R_1_ values in dCPu (**b**) vCPu (**c**) NAc (**d**) Ctx (**e**) PF (**f**) and HIP (**g**), and normalized optical density for TH in CPu (*n* = 6 mice). Numbers in the color-coordinated bar just above each plot indicate the A-P coordinate.

qAIM-MRI enabled activity mapping throughout the entire brain volume, and revealed abnormal brain function associated with pathological conditions. Significant correlations were observed between TH-immunoreactivity and R_1_ in the dorsal CPu (dCPu) at A-P = 0.25 mm and −0.5 mm (*P* < 0.05; Fig. 4b) and ventral CPu (vCPu) at A-P = 0.25 mm (*P* < 0.05; Fig. 4c). No significant correlations were observed in nucleus accumbens (NAc) (*P* > 0.05; Fig. 4d), or hippocampus, which has little relation to PD (*P* > 0.05; Fig. 4g). TH-immunoreactivity in the striatum has been correlated to motor-performance in MPTP-intoxicated mice^16^. Therefore, R_1_ in the CPu reflects the severity of PD. Region of the cortex (Ctx) at A-P = 0.25 mm (*P* < 0.05; Fig. 4e), and parafascicular nucleus of the thalamus (PF) (*P* < 0.02; Fig. 4f) also showed significant correlations between R_1_ and TH-immunoreactivity in the striatum.

## Discussion

Our results add further insights into AIM-MRI. First, Mn^2+^ accumulates in GABAergic neurons of the striatum depending on their neuronal activity; a relationship that was previously not analyzed quantitatively^1^. There are many reports that Ca^2+^ elevation is related to the firing activity in excitatory neurons^13^,^14^. Mn^2+^ can pass through opened Ca^2+^ channels including the voltage-dependent Ca^2+^ channels^3^,^4^; therefore, Mn^2+^ should accumulate depending on the activity of neurons regardless of whether they are inhibitory or excitatory neurons. The voltage-dependent Ca^2+^ channels and ionotropic glutamate receptors, including N-methyl-D-aspartic acid (NMDA) receptors, that are permeated by Mn^2+^ as well as Ca^2+^, are also expressed in astrocytes. Although these mechanisms have a minor role in astrocyte Ca^2+^ signaling *in situ*^18^,^19^, astrocytes are able to respond to presynaptic transmitter release, and Ca^2+^ influx occurs in response to neuronal activity (Supplementary Fig. S2). This means that in the presence of Mn^2+^ in the extracellular space, Mn^2+^ accumulation in astrocytes may correlate with the activity of adjacent neurons. Even assuming that not all types of cells accumulate Mn^2+^ depending on the neuronal activity, R_1_ in a tissue should relate to the neuronal activity in the region of interest (ROI) if some types of cells in the ROI accumulate Mn^2+^ depending on the neuronal activity. Thus, AIM-MRI can assess relative levels of neuronal activity.

Second, our qAIM-MRI method enables quantitative neural activity mapping of animal disease models compared with their healthy counterparts. Animal models of brain disease, including those for PD, are being increasingly investigated with MEMRI^1^,^20^,^21^, where Mn^2+^ was injected directly into brain nuclei, and alterations in connectivity or axonal transport in PD was examined^20^,^21^. We administered MnCl_2_ intraperitoneally without breaking the blood-brain barrier, which ensures slow and uniform administration to the entire extracellular brain space, making it possible to record the history of neuronal activity over the entire brain volume in awake, freely moving animals^7^; whereas, blood-oxygen-level dependent (BOLD) functional MRI, which relies on blood hemodynamics, can record the activity only in the MRI scanner, and positron-emission tomography (PET) or single-photon emission computed tomography (SPECT) can measure metabolic and neurochemical, for example DAergic, changes in the brains^22^, but cannot directly detect neuronal activities. Thus, our qAIM-MRI with quantitative T_1_ measurement enables the quantitative neuronal activity mapping over the entire brain volume, and can reveal how and where activities change in animal disease models, including PD.

Our qAIM-MRI results also offer insights related to PD. Although there are electrophysiological studies of the striatum in animal models of PD^23^,^24^,^25^,^26^, its pathophysiological role has been argued^9^,^10^,^11^. Larger Mn^2+^ accumulations in the dCPu of MPTP than of control mice indicated that striatal activity was elevated in PD (Fig. 2), in agreement with previous electrophysiological results^23^,^25^,^26^. Moreover, there are good correlations between R_1_ in the CPu, especially in dCPu, a sensorimotor region^27^, and TH-immunoreactivity in the striatum, which is related to the severity of PD in animal models^16^, suggesting that neuronal activity in the sensorimotor part of the striatum is associated with the severity of PD. DA excites striatal direct-pathway neurons via dopamine D1 receptors, and also inhibits striatal indirect-pathway neurons via D2 receptors; thus, the net effects of DA-loss may be no changes. Indeed, the mean firing rate decreased at direct pathway neurons and increased at indirect pathway neurons after 6-hydroxydopamine (6-OHDA) lesions^24^. However, spontaneous firing rate in the striatum, without distinguishing subtypes of neurons, increased after 6-OHDA lesions^25^,^26^. These phenomena may arise for the following reasons: (i) The mean firing rate of direct-pathway neurons is very low compared with indirect-pathway neurons in control rodents, and the increment in the firing rate in the indirect-pathway neurons after 6-OHDA lesion is much larger than the decrement in the direct-pathway neurons^23^,^24^; thus overall activity may increase after DA-loss in the striatum. (ii) Direct-pathway neurons express low affinity D1 dopamine receptors, while indirect-pathway neurons express high affinity D2 receptors^28^. Therefore, loss of DA may more effectively influence the indirect-pathway neurons. (iii) Medium spiny neurons exhibit oscillatory bursting after 6-OHDA lesions^29^. Bursting activity causes larger influxes of Ca^2+^ than does sporadic firing^13^,^14^,^30^. Thus, resulting in the increased accumulation of Mn^2+^ we observed in the dorsal striatum of the PD mice. Nonetheless, it would be useful to clarify which types of neurons are responsible for increasing R_1_ values in the striatum.

According to the classical model of the basal ganglia, dopamine depletion leads to increased activity in the indirect-pathway and reduced activity in the direct-pathway. This is thought to result in excessive inhibitory output to the thalamus. Thus, we also analyzed the region of the substantia nigra pars reticulata (SNr), which is the output nuclei of the basal ganglia, and the globus pallidus pars externa (GPe), which receives the input from the indirect-pathway neurons in the striatum (note: we did not analyze the region of the globus pallidus pars interna (GPi) or the subthalamic nucleus (STN), because they are too small to determine the region precisely on the MR image). SPM analyses revealed no significant differences between those T_1_ values in PD mice and control mice (Fig. 2). There were significant correlations between R_1_ values in SNr and GPe using Pearson’s correlation coefficients; however, no significant regression coefficients were detected in the regions by bootstrap 95% confidence limits after 1,000 randomizations (data not shown). These observations may be explained by reports showing no significant changes in the mean firing rates in the GPe^25^, GPi^25^, and SNr^31^.

Regions exhibiting significantly reduced T_1_ values were observed in the cortex and thalamus (Fig. 2), and significant correlations between TH-immunoreactivity and R_1_ values were observed in the sensorimotor area of cortex (Ctx) and the parafascicular nucleus of the thalamus (PF) (Fig. 4). DA depletion increases burst-firing in primary motor cortex, as well as the percentage of time spent in burst activity^28^, which may also help to explain our results. Systemic administration of either MPTP or 6-OHDA induces the selective degeneration of PF; however, the activity increases in the remaining PF neurons^32^, which may account for the reduced T_1_ value we observed in PF. The striatum receives glutamatergic input from areas of Ctx and PF; therefore, increased activity in Ctx and PF may lead to increased activity in the striatum. However, those findings are not consistent among studies; thus, further investigations are needed for clarifying the activity changes we observed in the cortex and the thalamus in PD animals.

Although, in general, TH expression in the striatum is decreased in PD model animals^16^,^17^,^33^ and PD patients^34^,^35^, the quantitative relationship between the expression of TH and the symptoms of PD had not been fully established. Therefore, we should more fully investigate the relationship between the R_1_ values in the striatum and PD symptoms like motor and gait performances in our PD model mice.

Mn^2+^ itself may alter neuronal activity by a variety of mechanisms, but these alterations are caused by concentrations of Mn^2+^ > 200 μM^36^,^37^,^38^. The R_1_ values we obtained after MnCl_2_ administration were less than 0.35 in the ventricular region (Supplementary Fig. S5) and less than 0.68 in the brain parenchyma (Fig. 4). These R_1_ values corresponded to less than 80 μM Mn^2+^ (Supplementary Fig. S3). This range of Mn^2+^ does not alter the neuronal activities. Indeed, there were no alterations in the condition of mice after MnCl_2_ administration compared with before administration. Moreover, in this study, R_1_ values in PD model mice were compared with those of their healthy counterparts administered the same amount of MnCl_2_. Thus, the differences in R_1_ values between the control and the PD mice were not caused by the effects of Mn^2+^ on neuronal activities.

There is currently no definitive diagnostic test for PD; patients are currently diagnosed based on clinical criteria scaled by psychomotor symptoms such as the Unified Parkinson’s Disease Rating Scale (UPDRS)^12^,^39^. However, these motor and non-motor symptoms are insufficient for distinguishing PD from other diseases^12^. A conclusive method of early diagnosis would be of great value for appropriate treatment. qAIM-MRI quantification of R_1_ in dCPu enables a diagnosis of the severity of PD to ensure appropriate treatment, as long as Mn^2+^ toxicity is avoided with a manganese chelator^1^.

This work establishes a foundation for the extension of MEMRI techniques to studying animal models of brain disease. Because qAIM-MRI can be used for non-invasive investigation of whole brain activity that does not depend on blood hemodynamics but directly on neuronal activity^1^,^7^, our findings pave the way for significant progress in research on PD pathophysiology, and suggest that qAIM-MRI can be utilized not only for diagnosing PD, but also potentially for the study and diagnosis of various other neurological disorders.

## Methods

### Mice

For the Ca^2+^ imaging study to discriminate striatal GABAergic neurons, we used heterozygous GAD67-GFP knock-in mice (GAD67-GFP mice), in which enhanced-GFP is selectively expressed under the control of the endogenous GAD67 gene promoter^40^. The colony was maintained by crossing male GAD67-GFP mice with female C57BL/6 mice (Clea Japan). For the MRI and immunohistochemical study, we used male C57BL/6 mice. All mice were housed and maintained at 22–24 °C on a 12-h light/dark cycle and permitted *ad libitum* access to food and water. The Tohoku University Committee for Animal Experiments approved all animal experiments, and the experiments were performed in accordance with the Guidelines for Animal Experiments and Related Activities of Tohoku University, as well as the guiding principles of the Physiological Society of Japan and the National Institutes of Health (NIH), USA.

### Quantification of intracellular Ca^2+^ elevation and Mn^2+^ accumulation

To discriminate GABAergic neurons, we used corticostriatal slices prepared from GAD67-GFP mice, which express the GFP in GABAergic neurons including the projection neurons and the interneurons^40^, as previously described^41^,^42^. Briefly, postnatal day 21 (P21) to P23 GAD67-GFP mice of either sex were anesthetized with isoflurane (Mylan) and decapitated. The brain was rapidly isolated and placed in ice-cold artificial cerebrospinal fluid (ACSF) bubbled with 95% O_2_–5% CO_2_. The composition of ACSF was as follows (in mM): 137 NaCl, 2.5 KCl, 0.58 NaH_2_PO_4_, 1.2 MgCl_2_, 2.5 CaCl_2_, 21 NaHCO_3_, and 10 glucose. Corticostriatal sagittal slices (300 μm thick) were prepared using a vibratome tissue slicer (VT-1200S, Leica Microsystems) and incubated at room temperature in a submerged chamber containing gassed ACSF for at least 60 min prior to the experiments.

[Ca^2+^]_i_ elevation and Mn^2+^ accumulation were measured in striatal cells loaded, as previously described^41^,^42^, with the ratiometric Ca^2+^ sensitive dye Fura-2 LR/AM (Calbiochem). To identify astrocytes, 1 μM sulforhodamine 101 (SR101, Sigma) was dissolved in dye-loading solution^43^. After dye-loading, the slice was transferred to a continuously superfused (2–2.5 ml/min) chamber, and the fluorescence was observed by an epifluorescence upright microscope (BX51WI, Olympus) equipped with a 20×, NA 1.0 water-immersion objective (Olympus). The Fura-2 LR-loaded slices were excited at wavelengths of 360 or 380 nm using a filter changer (Lambda DG-4, Sutter Instruments), and fluorescent signals at 510 nm were captured (F360 and F380) with an EM-CCD camera (DU-885 or DU-897, Andor Technology). The [Ca^2+^]_i_ transients or Mn^2+^ accumulations were evoked by stimulation with 200-μs, 200-μA biphasic current pulses at various frequencies from a glass microelectrode (tip diameter, ~30 μm) placed on the corticostriatal fiber tract. All equipment was controlled by iQ software (Andor Technology). The experiments were performed under temperature control (30 ± 1 °C).

The analysis of the imaging data was performed with ImageJ software^44^ and custom-made programs written in MATLAB (MathWorks). We identified GFP-positive cells (i.e. GABAergic neurons), and measured the average fluorescence (F360 and F380) within the region of interest (ROI) of these cells as a function of time. [Ca^2+^]_i_ transients in a striatal cell were estimated by the fluorescence ratio (R = F360/F380) from each imaged cell. The frame rate was 8–10 frames per second (fps). The baseline was set to the mean R-value in 10 frames just before stimulation, and the change in the R-value from the baseline was defined as ΔR. To compare the Ca^2+^ transients among the stimulus frequencies (Supplementary Fig. S1), fast imaging was needed. The frame rate was 17–20 fps. The relative changes in [Ca^2+^ ]_i_ were quantified as −ΔF/F at 380 nm, instead of ΔR, where F indicates the mean fluorescence intensity in 10 frames before stimulation, and ΔF is the change in fluorescence intensity from F. The peak value of ΔR or −ΔF/F after the stimulation was used for the amplitude of the [Ca^2+^]_i_ transient.

Mn^2+^ quenches the Fura-2 LR fluorescence emission^45^. Thus, for quantification of intracellular Mn^2+^ accumulation, the amount of the quench was quantified as −ΔF/F at 360 nm. The mean value of −ΔF/F within 10 frames beginning 4 s after the stimulation was used for the amount of the quench.

### MPTP treatment

For the MRI study preceding immunohistochemistry, batches of 3–6 mice were used to ensure reproducibility. The mice in each batch were randomly assigned to two groups. Those in the first group were injected with 1-methyl-4-phenyl-1,2,3,6-tetrahydropyridine hydrochloride (MPTP, Sigma; 20 mg kg^–1^ in saline, i.p.) four times at 2-h intervals^15^ (MPTP group, *n* = 7). Those in the second group were treated simultaneously and identically with saline only instead of MPTP (control group, *n* = 7). One mouse that succumbed to the MPTP injection was excluded from the MPTP group. MRI and immunohistochemical experiments were performed blindly (the experimenters did not know which mice were injected with MPTP or saline).

### Activation-induced, manganese-enhanced magnetic resonance imaging (AIM-MRI)

One or two weeks after MPTP treatment, mice in both the MPTP and control groups were injected with MnCl_2_ solutions (0.2 mmol kg^–1^ in saline, i.p.) twice at 24-h intervals^7^. None of the mice succumbed to the MnCl_2_ administration. Because excess extracellular Mn^2+^ would shorten T_1_ and mask the alteration of T_1_ due to intracellular Mn^2+^ accumulation, MRI acquisition must be conducted after clearance of Mn^2+^ from the extracellular space. Thus, we analyzed the time course of R_1_ (=T_1_^–1^) in the ventricle of control mice after MnCl_2_ injection (Supplementary Fig. S5). R_1_ values 5 h and 24 h after the last MnCl_2_ injection (0.343 ± 0.012 s^−1^, *n* = 12, *P* < 0.001 at 5 h; 0.324 ± 0.009 s^−1^, *n* = 14, *P* < 0.05 at 24 h) were significantly higher than that before injection (0.288 ± 0.006 s^−1^, *n* = 10), and gradually returned to the pre-injection level; there was no significant difference between the R_1_ before MnCl_2_ injection and that more than 48 h after injection (0.315 ± 0.007 s^−1^, *n* = 15 at 48 h, *P* > 0.05). R_1_ in the striatal parenchyma 48 h after the last MnCl_2_ injection (0.544 ± 0.011 s^−1^, *n* = 6) was significantly different from that before injection (0.436 ± 0.007 s^−1^, *n* = 6, *P* < 0.05; data not shown). In consideration of these factors, the time point for qAIM-MRI acquisition was chosen to be 48 h after MnCl_2_ administration. One control mouse that exhibited a T_1_ value less than 3 s in the ventricle before the MnCl_2_ administration was excluded from further analysis.

Before and 48 h after MnCl_2_ administration, MRIs were acquired (Supplementary Fig. S3) using an AV 400 WB 9.4-T, 89 mm spectrometer equipped with a 45 G/cm gradient insert (Bruker BioSpin). A 38-mm 1H volume coil (Bruker BioSpin) was used for transmission and signal detection. After pre-anesthesia in a pre-anesthesia box with a mixture of air and O_2_ (air:O_2_ = 8:2) containing 2–3% isoflurane (Mylan), mice were positioned into the MRI scanner and maintained at 1–2% isoflurane using a nose cone during the scanning. Body temperature was maintained by a circulation of heated water. For T_1_ measurement of the brain, rapid acquisition with relaxation enhancement (RARE) with variable repetition time (RARE-VTR) pulse sequence with 7 TR values (450, 600, 900, 1,500, 2,500, 4,500, and 7,500 ms) was used with effective echo time (TE_eff_) = 8.1 ms, matrix size = 128 × 128, field-of-view (FOV) = 1.6 × 1.6 cm^2^, slice thickness = 0.5 mm, and number of slice = 20. Multislice, fast spin-echo T_2_-weighted images (RARE, TE_eff_ = 22 ms, TR = 2,500 ms) were acquired and used to co-register images to the mouse brain template. Total time in the MRI scanner for mice was about 30 min, and they were then returned to their home cage.

For confirmation of the correlation between Mn^2+^ concentrations ([Mn^2+^]) and longitudinal relaxation rates (R_1_), phantoms of 2% (wt/vol) agarose gel (NuSieve 3:1, Lonza) dissolved in artificial intracellular solution (140 mM K-methanesulfonate, 2 mM NaN_3_, and 20 mM HEPES-Na (pH ~7.2)) with different amounts of MnCl_2_ (0, 20, 40, 60, 80 and 100 μM) were imaged by the sequence for T_1_ measurement described above.

### MR image analysis

Parametric T_1_ maps were calculated pixel-by-pixel by fitting using ParaVision 5.1 software (Bruker BioSpin). After spatial filtering, the theoretical expression of the signal intensity (SI) in each pixel:


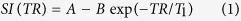


was fitted to experimental data. Pixels in which T_1_ values were longer than 4,000 ms or shorter than 500 ms were excluded from the analysis. Co-registration of T_1_ maps to the Allen Reference Mouse Brain Atlas^46^ (2014 Allen Institute for Brain Science. Allen Mouse Brain Atlas, http://mouse.brain-map.org/) was performed as follows: 1) A T_2_-weighted mouse brain template image was acquired by 3D-RARE sequence with TE_eff_ = 45 ms, matrix size = 256 × 256 × 256, field-of-view (FOV) = 2.2 × 2.2 × 2.2 cm^3^. 2) The T_2_-weighted images were registered to the T_2_-weighted template image, and the T_1_ maps were co-registered simultaneously using SPM8 software (Wellcome Trust Centre for Neuroimaging, University College London). 3) The mouse brain atlas was registered to the T_2_-weighted template image manually using pMod software (PMOD Technologies). We could then determine the regions of interest by querying structures from the brain atlas. The origin coordinate was determined at the midline in the medial-lateral (M-L) direction, the vertex of the cerebral cortex in the dorsal-ventral (D-V) direction, and bregma in anterior-posterior (A-P) direction.

The co-registered T_1_ maps were smoothed with a Gaussian kernel with FWHM 0.25 mm in the x-y plane and 0.5 mm in the z-axis, and an unpaired Student’s *t*-test was used to determine which voxels decreased or increased in T_1_ in the MPTP group compared with the control group using SPM8. A parametric map of voxels with statistically significant changes in T_1_ was created and was overlaid on the T_2_-weighted template image.

For ROI analysis, brain structures of interest were extracted from the atlas and superimposed over the T_1_ maps of each mouse. The region of the CPu was divided into two subregions, somatosensory region (dCPu) and associative/limbic region (vCPu)^27^. The mean T_1_ values in the ROIs were used then for analysis.

### Immunohistochemistry

After T_1_ measurement (11 to 20 d after MPTP administration; Supplementary Fig. S3), mice were sacrificed by cervical dislocation and transcardially perfused with ~30 ml of saline followed by ~30 ml of 4% (wt/vol) paraformaldehyde in phosphate buffered saline (PBS, pH 7.4). Brains were then fixed overnight in the same solution at 4 °C, and then re-suspended in 10% (wt/vol) sucrose in PBS followed by 20 and 30% (wt/vol) sucrose for cryo-sectioning. Cryosections were cut at a thickness of 20 μm and stored in PBS containing 0.05% (wt/vol) NaN_3_ until use. Immunostaining was carried out with free-floating brain sections^47^.

For the histological detection of tyrosine hydroxylase (TH)-positive fibers in striatum, the sections were immunohistochemically stained with anti-TH antibody (MILLIPORE, 1:10,000) using the ABC method (Vectastain elite ABC kit, Vector Laboratories), according to the supplier’s recommendations. Briefly, the sections were incubated in PBS containing 0.3% (vol/vol) H_2_O_2_ for 15 min to inhibit endogenous peroxidase activity, pre-incubated with 3% (wt/vol) BSA in PBS containing 0.3% (vol/vol) Triton X-100 for 1 h, and incubated with anti-TH antibody in PBS containing 3% (wt/vol) BSA and 0.3% (vol/vol) Triton X-100 overnight at room temperature. The sections were then incubated with biotinylated secondary antibody for 1 h, followed by avidin-biotin-peroxidase complex for 30 min at room temperature. Finally, the sections were reacted with 3,3′-diamino benzidine (DAB) using a Vector DAB substrate kit (Vector Laboratories) for color development. For detection and observation, a microscope (BX51, Olympus) was used at a magnification of 12.5×.

For the histological detection of TH-positive neurons in the substantia nigra pars compacta (SNc) and ventral tegmental area (VTA), we used double immunofluorescence staining with anti-TH (1:10,000) and anti-neuronal nuclei (NeuN) antibodies (MILLIPORE, 1:5,000). The sections were pre-incubated with 3% (wt/vol) BSA in PBS containing 0.3% (vol/vol) Triton X-100 for 1 h. They were then incubated with primary antibodies in PBS containing 3% (wt/vol) BSA and 0.3% (vol/vol) Triton X-100 overnight at room temperature. The sections were then incubated with secondary antibodies (anti-mouse or anti-rabbit IgG conjugated with Alexa Fluor 488 or 546, Invitrogen, Carlsbad, CA, 1:200) for 1 h at room temperature. For detection and observation, a fluorescent microscope (BH2, Olympus) was used at a magnification of 200×. Analysis of the images was performed using computer-associated image analyzing software (WinRoof Version 5, Mitani Corporation), as described previously^48^,^49^,^50^.

### Statistical analysis

Statistical analyses were performed using JMP Pro 11 (SAS Institute), MATLAB, and SPM8 software. Statistically significant differences (*P* < 0.05) were assessed by the Mann-Whitney U test, Wilcoxon signed rank test, Kruskal-Wallis test with Tukey-Kramer post-hoc test, or Friedman test with Tukey-Kramer post-hoc test for comparisons between the mean values of two unpaired groups or paired groups and multiple comparisons with unpaired values or paired values, respectively. For the statistical parametric mapping (SPM) analysis, the statistical significance (*P* < 0.025) was assessed based on unpaired Student’s *t*-test using SPM8 software. To ascertain the correlation between two variables, we employed the Pearson’s correlation coefficient, and if a significant correlation (*P* < 0.05) was detected, we confirmed the significance of the regression coefficient by bootstrap 95% confidence limits after 1,000 randomizations. *P*-values are two-sided. All data are presented as mean ± s.e.m., unless stated otherwise.

## Additional Information

**How to cite this article**: Kikuta, S. *et al.* Quantitative activation-induced manganese-enhanced MRI reveals severity of Parkinson's disease in mice. *Sci. Rep.* **5**, 12800; doi: 10.1038/srep12800 (2015).

## Supplementary Material

Supplementary Figure S1-S5
