# Supplementary material for: Quantitative activation-induced manganese-enhanced MRI reveals severity of Parkinson’s disease in mice
Source: Sci Rep. 2015 Aug 10;5:12800. doi: 10.1038/srep12800 (PMC4530460; doi:10.1038/srep12800)
Supplement: Supplementary Figure S1-S5 [file srep12800-s1.pdf]

Supplementary Information

Supplementary Figure S1-S5

Quantitative activation-induced manganese-enhanced MRI reveals severity of Parkinson's disease in mice

Authors: Satomi Kikuta[1, 2, 3], Yukiyo Nakamura[4], Yukio Yamamura[4], Atsushi Tamura[1, 2], Noriyasu Homma[1], Yuchio Yanagawa[2, 5], Hajime Tamura[1], Jiro Kasahara[4] & Makoto Osanai[1, 2*]

Affiliations:

[1]Tohoku University Graduate School of Medicine, 2-1 Seiryo-machi, Aoba-ku, Sendai 980-8575, Japan.

[2]CREST, Japan Science and Technology Agency, 4-1-8 Honcho, Kawaguchi 332-0012, Japan

[3]Research Fellow of the Japan Society for the Promotion of Science.

[4]Graduate School and Faculty of Pharmaceutical Sciences, Institute of Biomedical Sciences, Tokushima University, 1-78 Shoumachi, Tokushima 770-8505, Japan.

[5]Gunma University Graduate School of Medicine, 3-39-22 Showa-machi, Maebashi 371-8511, Japan.

*Correspondence should be addressed to M.O. (osanai@med.tohoku.ac.jp)

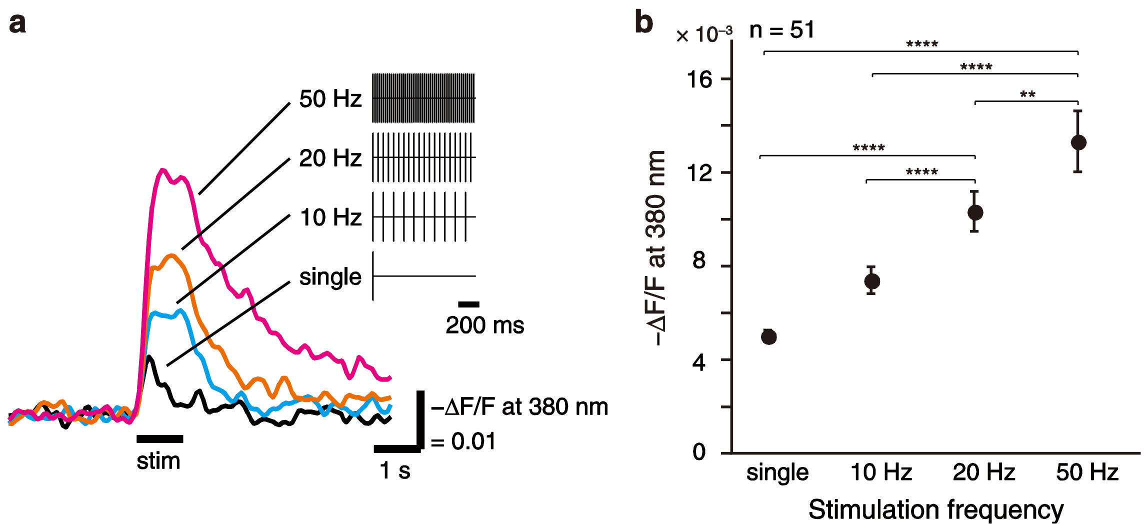

Supplementary Figure S1

The amplitude of [Ca^{2+}]$_i$ transient was a function of neuronal activity.

(a) Typical time courses of the [Ca^{2+}]$_i$ transients evoked by a single pulse (black line), and 1 s of 10-Hz (light blue), 20-Hz (orange), and 50-Hz (magenta) stimulations. The stimulus period for the 10-50 Hz stimulations is shown by a horizontal black bar just above “stim” and the single stimulation was applied at the left end of the bar. Schema of the stimulation patterns are shown at upper right. **(b)** Amplitudes of the fluorescence changes at 380 nm (−ΔF/F at 380 nm) are plotted against stimulation frequencies (n = 51 cells). **$P < 0.01$, ****$P < 0.001$.

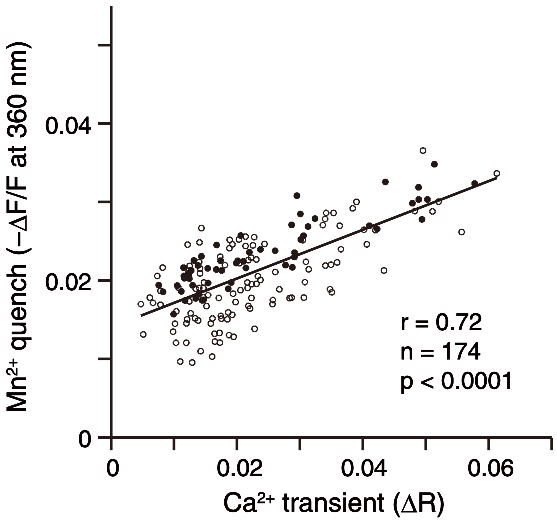

Supplementary Figure S2

Intracellular Mn^{2+} accumulation was correlated with Ca^{2+} elevation in astrocytes.

Comparison of the amplitude of the $[Ca^{2+}]_i$ transient and the amount of Mn^{2+} quench of the fluorescence at 360 nm in SR101-positive cells, putative astrocytes, in striatum when 20 pulses at 20 Hz (solid circle) or 50 Hz stimuli (open circle) were applied (n = 174 cells). r: Pearson's correlation coefficient.

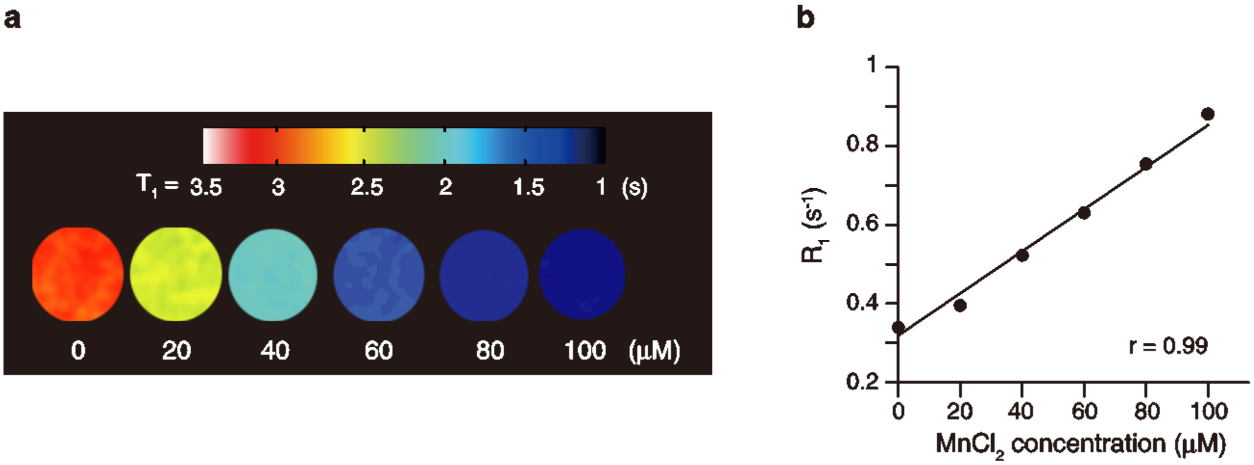

Supplementary Figure S3

T_1 is a function of Mn^{2+} concentration.

(a) Pseudo color T_1 images of the 2% agarose gel phantoms with various concentrations of $MnCl_2$ (indicated just below each image). T_1 maps were calculated pixel-by-pixel as described in Materials and Methods. **(b)** R_1 (= $1/T_1$) values are plotted against $MnCl_2$ concentration. A highly significant linear correlation was observed between the two (n = 4 MRI acquisitions, $P < 0.0001$, r: Pearson's correlation coefficient); thus, T_1 was inversely proportional to Mn^{2+} concentration. Data points are means ± s.e.m. (error bars are too small to see).

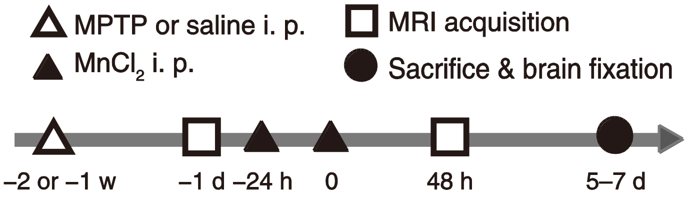

Supplementary Figure S4

Experimental design.

The schedule for MPTP administration, $MnCl_2$ injection, MRI acquisition, and fixation are indicated. w: week.

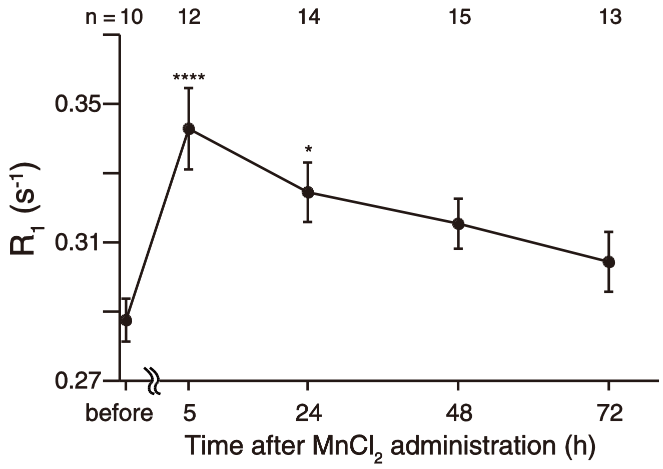

Supplementary Figure S5

Time course of R_1 in the ventricle after $MnCl_2$ administration.

R_1 values are plotted against the time after last $MnCl_2$ administration. R_1 values 5 h and 24 h after last $MnCl_2$ injection were significantly different from that before $MnCl_2$ injection. ****P < 0.001, *P < 0.05. Data were obtained from 3 mice and *n* indicates the number of ROIs placed at the ventricle region.
